# Primary Sjögren’s syndrome with renal tubular acidosis and central pontine myelinolysis: An unusual triad

**DOI:** 10.5414/CNCS110994

**Published:** 2023-05-05

**Authors:** Zibya Barday, Malcolm Masikati, Nicola Wearne, Brian Rayner, Bianca Davidson, Kathleen Jane Bateman, Erika Jones

**Affiliations:** 1 Department of Medicine,; 2 Division of Nephrology and Hypertension, Kidney and Hypertension Research Unit, and; 3 Division of Neurology, Groote Schuur Hospital, University of Cape Town, Cape Town, South Africa

**Keywords:** Sjögren’s, kidney, renal tubular acidosis, central pontine myelinolysis

## Abstract

Primary Sjögren’s syndrome (pSS) is a complex, multisystem autoimmune disorder. It is characterized by lymphocytic infiltration of the exocrine glands. In the setting of pSS, the presence of systemic disease is an important prognostic determinant, but involvement of the kidney is uncommon. The triad of pSS, distal renal tubular acidosis (dRTA), and central pontine myelinolysis (CPM) is rare and potentially fatal. A 42-year-old woman presented with dRTA, profound hypokalemia, and CPM characterized by progressive global quadriparesis, ophthalmoplegia, and encephalopathy. Sjögren’s syndrome was diagnosed based on sicca symptoms, clinical features, and strongly positive anti-SSA/Ro and anti-SSB/La autoantibodies. The patient responded well to electrolyte replacement, acid-base correction, corticosteroids, and subsequent cyclophosphamide therapy. Early recognition and appropriate treatment resulted in good kidney and neurological outcomes in this case. This report highlights the need to consider the diagnosis of pSS in unexplained dRTA and CPM, as it has a favorable prognosis if recognized and managed timeously.

## Introduction 

Primary Sjögren’s syndrome (pSS) is a multisystem autoimmune disorder characterized by lymphocytic infiltration of the exocrine glands, predominantly the salivary and lacrimal glands [1]. pSS is the second most common rheumatological condition, following rheumatoid arthritis [2]. It has an estimated the prevalence between 0.2 and 0.8% [3]. Literature on the prevalence of pSS in Africa is scarce, however, the reported frequency in studies ranges between 1.8% and 47.6% [4]. Classically, it occurs in women (female : male ratio 9 : 1), between the fourth and fifth decade of life. 

The diagnosis of pSS utilizes the 2016 American College of Rheumatology/European League Against Rheumatism (ACR/EULAR) criteria, which incorporates a combination of clinical and laboratory characteristics [5]. The EULAR recommends screening people with pSS for kidney disease utilizing the EULAR Sjögren’s syndrome disease activity index (ESSDAI) [6]. The presence of anti-Sjögren’s-syndrome-antigen B (anti-SSB) antibodies and rheumatoid factor may be associated with more systemic disease, including kidney involvement [6]. In pSS, kidney manifestations are unusual but important to recognize. It is estimated that 5% of pSS have kidney involvement [7]. However, neurological manifestations are more common. Central pontine myelinolysis (CPM) is a rare, severe neurological manifestation of pSS. Several case reports have demonstrated pontine lesions, which are usually reversible [8, 9, 10]. 

In pSS, the presentation of distal renal tubular acidosis (dRTA) together with CPM is rare and potentially fatal. This report highlights the need to consider a diagnosis of pSS in unexplained dRTA and to be aware of the neurological manifestations, such as CPM, which potentially have a favorable outcome if managed timeously. 

## Case report 

A 42-year-old woman presented to her general practitioner with a 3-month history of fatigue, myalgia, and muscle weakness. She had an excellent functional baseline, with well-controlled hypertension and asthma. She was of sober habits. It was suspected that she had had COVID-19 infection, despite a negative PCR, and was treated with a short course of oral prednisone (40 mg for 10 days). During this period there was mild improvement in her symptoms. After completing the course of prednisone, her symptoms deteriorated. This resulted in an emergency unit admission with profound generalized muscle weakness and respiratory hypoventilation requiring intubation and ventilation. On examination, the rest of her vital signs were normal. Her physical examination revealed a normal body habitus, profound motor weakness (0/5 power), with brisk reflexes, and up-going plantar reflexes. She had no encephalopathy and no ocular or bulbar signs. At presentation, she had profound hypokalemia (K^+^ 1.4 mmol/L) with a normal anion gap metabolic acidosis (pH 7.29, pCO_2_ 4.1kPa, HCO_3_ 16.2 mEq/L, BE –11.7mEq/L). She was transferred to an intensive care unit (ICU) for further management. 

Her medication included a thiazide diuretic (hydrochlorothiazide 12.5 mg orally, daily), angiotensin converting inhibitor (enalapril 2.5 mg orally, twice daily) and metered dose inhalers, with salbutamol and budesonide. There was no non-steroidal anti-inflammatory usage reported. 

**Figure 1.**
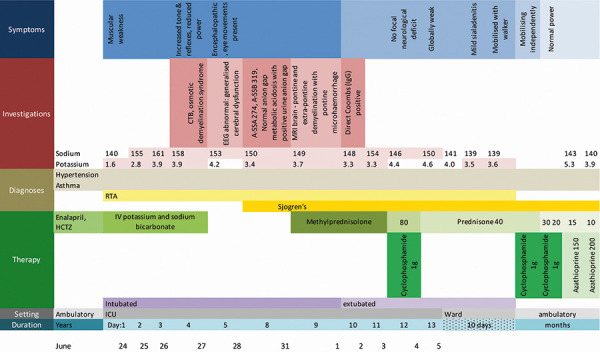
Sequence of events, including investigations, management, and response.

The sequence of events, including the laboratory results, are shown in Figure 1. Investigations revealed a normal anion gap metabolic acidosis (anion gap 15.8 mmol/L) and a positive urinary anion gap (79.2 mmol/L), in keeping with a diagnosis of a dRTA. The serum osmolality was calculated at 288 mmol/kg, and the urine osmolality was 286 mmol/kg. The patient received intravenous potassium chloride as well as oral and intravenous 8.3% sodium bicarbonate to correct the hypokalemia and the metabolic acidosis. Shortly after admission, her serum Na^+^ increased from 140 to 155 mmol/L, which was attributed to poor urinary concentration related to profound hypokalemia, in addition to hypertonic sodium bicarbonate solution administration. The presenting urea and creatinine were 10.2 mmol/L and 103 μmol/L, respectively, but settled to 7.4 mmol/L and 66 μmol/L on discharge. An ultrasound showed normal kidney sizes and normal echogenicity, with no calcification or stones visualized. The diagnosis of pSS was made on the basis of a positive antinuclear antibody with a titre of 17.0; positive anti-SSA/Ro autoantibody (274.0 U/mL); positive anti-SSB/La autoantibody (319.0 U/mL) and hypergammaglobulinemia. There was no salivary gland biopsy performed at the time. 

**Figure 2.**
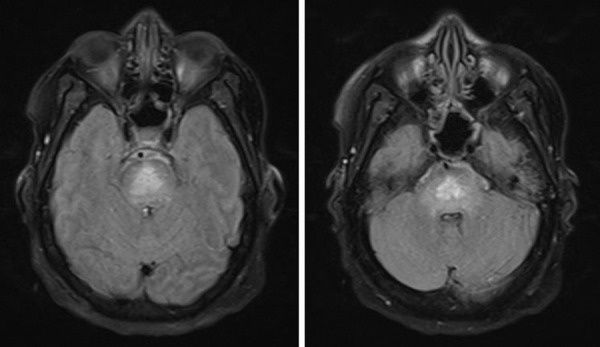
MRI T2-weighted images illustrating a focal triangular hyperintense lesion in the pons suggestive of central pontine myelinolysis.

Despite fluid and electrolyte correction, the patient developed encephalopathy with ophthalmoplegia and worsening quadriparesis. On day 10 of admission, a magnetic resonance imaging (MRI) was performed (Figure 2). The MRI revealed focal hyperintensity in the pons, suggestive of CPM, which was suspected to be due to a pSS in addition to severe hypernatremia, with significant serum sodium shifts. The lumber puncture results revealed an inflammatory lymphocytosis, with elevated IgG index and oligoclonal bands. There was no EMG performed at the time. 

The patient was treated with methylprednisone 1 g intravenously daily for 3 days, followed by 40 mg prednisone daily. She was also treated with cyclophosphamide 1 g intravenously monthly for 3 months, followed by maintenance azathioprine. She had a significant improvement in her mental state, and her muscle power returned to normal over 24 – 48 hours. She was safely extubated and discharged to the general medical ward. Post-extubation, she reported a 3-month history of pre-existing sicca symptoms. Keratoconjunctivitis sicca was confirmed by an ophthalmologist, and lubricants were prescribed. 

## Discussion 

This is a rare case of pSS characterized by dRTA and CPM, presenting with profound hypokalemia, progressive global quadriparesis, ophthalmoplegia, and encephalopathy. Although this case does not reflect the prototypical case vignette of CPM, which occurs in the setting of severe chronic hyponatremia with a rapid serum sodium correction (greater than 12 mEq/L in 24 hours), the iatrogenic sodium shifts in this case were significant. This case illustrated a normal serum sodium at index presentation (140 mmol/L), with subsequent increase in serum sodium greater than 12 mmol/L over 24 hours. Importantly, upper motor neuron signs, which are not in keeping with hypokalemia, pre-dated the changes in serum sodium. The MRI findings, in addition to the inflammatory cerebrospinal fluid (CSF) results, suggest pSS brainstem involvement. Treatment with aggressive intravenous sodium bicarbonate requires careful monitoring due to the risk of CPM with rapid serum sodium changes. This case highlights the complexity of pSS as a multisystem disease. Furthermore, in the setting of unexplained dRTA, a diagnosis of pSS should be considered. 

**Figure 3.**
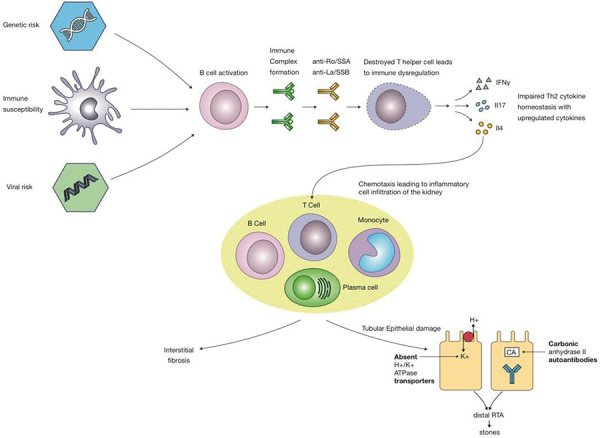
Pathogenesis of interstitial nephritis and renal tubular acidosis in primary Sjögren’s syndrome.

In pSS, lymphocytic infiltration can affect multiple organs. Extra-glandular manifestations are reported in 15% of cases [11]. Presentation with kidney disease is often heterogeneous. Clinical symptoms are often insidious and can precede sicca symptoms. Acute or chronic tubulointerstitial nephritis (TIN) is the most common histological pattern, accounting for 85% of cases involving the kidneys [12]. The clinical manifestations may range from tubular dysfunction, with or without acute kidney injury, to slowly progressive decline in kidney function [13]. TIN can occur prior to the onset of sicca symptoms, so pSS should be considered in patients with TIN, renal tubular acidosis (RTA), and hypokalemia [14]. The pathogenesis leading to the development of interstitial nephritis in pSS is shown in Figure 3. 

In pSS, the incidence of dRTA varies widely and is reported in published case series to range between 5 and 70% [12, 14, 15, 16]. Hypokalemia is a common finding and occurs secondary to urinary potassium wasting. It is usually asymptomatic, but case reports have described rare presentations of flaccid paralysis and respiratory arrest [12, 17]. However, in the setting of hypokalemia, assessing urine osmolality is essential. In order to demonstrate that potassium losses are in the urine, urine osmolality needs to be low, coinciding with a high or normal urinary potassium. Furthermore, in the setting of nephrogenic diabetes insipidus due to chronic hypokalemia, electrolyte correction needs to be carefully monitored, as large quantities of hypertonic sodium bicarbonate can give rise to CPM. In pSS, the most frequent type of RTA is dRTA. The exact mechanism is unknown. There are studies revealing the absence of the H+/K+ATPase transporters or TIN autoantibodies directed against carbonic anhydrase II [18, 19]. Nephrolithiasis can occur secondary to the hypercalciuria and hypocitraturia accompanying the dRTA. Glomerular involvement is uncommon, with membranoproliferative glomerulonephritis secondary to cryoglobulinemia being the most frequently described [20]. 

**Figure 4. Figure4:**
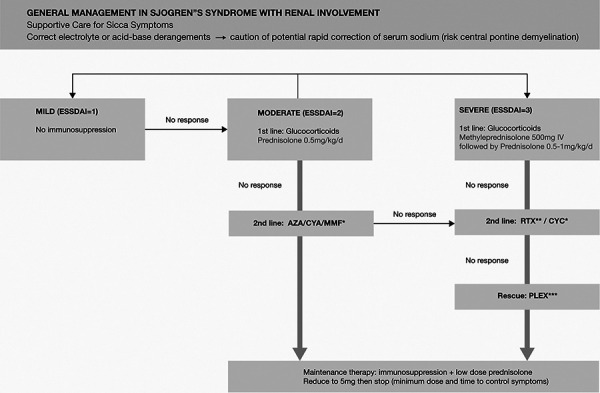
Management of primary Sjögren’s syndrome with renal involvement. AZA = azathioprine; CYA = cyclosporin; MMF = mycophenolate mofetil; RTX = rituximab; CYC = cyclophosphamide; PLEX = plasma exchange; ESSDAI = EULAR Sjögren’s syndrome Disease Activity Index. *No head-to-head comparison studies. **Cryoglobulin vasculitis. ***Life-threatening cryoglobulinemia vasculitis.

The frequency of neurological manifestations of pSS is estimated to be 20% [21, 22]. The neurological manifestations include poly- or mononeuropathies. Less frequently, the central nervous system (CNS) may manifest with a wide array of neurological presentations (e.g., focal deficits, meningoencephalitis, encephalopathy, myelopathy, optic neuritis, brainstem lesions, seizures, and mood disorders) [8]. However, CPM is a rare manifestation.[Fig Figure4]

Very few case reports describe kidney and CNS involvement as the primary presentation of pSS. As far as we are aware, 9 cases of pSS, dRTA, and CPM have been described (Table 1). All cases were female with age ranging between 17 and 75 years. Only 3 were known to have pSS prior to index presentation. In the majority, a combination of CPM and dRTA was present at their initial presentation. Only 3 of the 9 cases were associated with hypernatremia on presentation. The majority had positive anti-SSA/Ro antibodies. All cases were diagnosed on MRI, and all reported good neurological recovery on treatment with immunosuppression, within days to weeks, unlike those typically described in CPM from rapid correction of chronic hyponatremia. 

## Conclusion 

In conclusion, pSS is a complex autoimmune multisystem disorder. Extra-glandular manifestations, such as renal dRTA with CPM are rare and potentially fatal. This report highlights the need to consider a diagnosis of pSS in unexplained dRTA, the importance of careful monitoring of serum sodium shifts with intravenous therapies, and the fact that CPM has a favorable prognosis if recognized and managed early. [Table Table2]

## Human ethics 

Patient consent was obtained for the publication of this case study. 

## Funding 

There was no support/funding for this report. 

## Conflict of interest 

The authors declare that no competing interests exist in this case study. 

**Table 1.** Ten case series of primary Sjögren’s syndrome with renal tubular acidosis and central pontine myelinolysis.

	Bruns et al. [23]	Abdulla et al. [24]	Maturu et al. [8]	Nagashima et al. [25]	Saxena et al. [26]	KH Yoon et al. [10]	Watson et al. [27]	Rubo, S et al. [9]	Index Case
	Germany	India	India	Japan	India	Singapore	London	China	South Africa
Age	17	28	33	42	45	47	64	75	42
Sex	Female	Female	Female	Female	Female	Female	Female	Female	Female
Sicca symptoms	No	Yes	-	Yes	Yes	No	Yes	Yes	Yes
Diagnosis type I RTA	On index CPM admission	Occurred prior to CPM diagnosis	Occurred 1 year after CPM diagnosis	On index CPM admission	On index CPM admission	Occurred prior to CPM diagnosis	On index CPM admission	On index CPM admission	On index CPM admission
On presentation	Muscular weakness with leg pain. Later developed bilateral 6^th^ nerve palsies, dysarthria and dizziness; Additional Hashimoto thyroiditis, vitiligo, celiac disease	Jaundice, hepatic encephalopathy, generalized tonic clonic seizures; Additional hepatitis A infection	Sudden onset, rapidly progressive quadriplegia, severe dysarthria, bilateral facial palsies, bulbar palsy	Periodic weakness from hypokalemia for 9 years prior to index presentation	Progressive weakness of all 4 limbs, difficulty with respiration and swallowing, dysarthria and altered sensorium	Obtunded with quadriplegia; previous history of hypokalemic weakness and small vessel vasculitis of the bowel	3-month history of fatigue, nausea and vomiting, hypercalcemia; later developed bilateral 6^th^ nerve palsies with generalized weakness	Rapid, progressive quadriplegia, hypersomnia, dysphagia	Progressive global quadriplegia, ophthalmoplegia, and encephalopathy
Admission serum sodium	–	137 mmol/L	Hypernatremia; no history of hyponatremia with rapid sodium correction	–	Hypernatremia	Hypernatremia (154 mmol/L)	Reported normal	–	140 mmol/L
Admission serum potassium	1.8 mmol/L	2.6 mmol/L	2.2 mEq/L	Low	1.9 meq/L	Low	2.0 mmol/L	1.4 mmol/L	1.6 mmol/L
ANA	Positive	Positive	–	–	Positive	Positive	Positive	Positive	Positive
Anti SSA +/Anti SSB +	Anti SSA +/anti SSB +	Anti SSA +/anti SSB +	Anti SSA +/anti SSB +	Anti SSA +/anti SSB -	Anti SSA +/anti SSB -	Anti SSA +/anti SSB–	Anti SSA +/anti SSB–	Anti SSA +/anti SSB +	Anti SSA +/anti SSB +
Serum IgG and IgA	–	–	–	–	–	Elevated	–	–	Elevated
Salivary gland biopsy	–	–	Yes	Yes	–	–	–	–	No
Treatment	Electrolyte and acid-base balance correction, steroids	Electrolyte and acid-base balance correction	Electrolyte and acid-base balance correction, steroids	–	Electrolyte and acid-base balance correction, steroids, CYC	Steroids, CYC, and IVIG; After relapse required PE and IVIG	Electrolyte and acid-base balance correction, hydroxychloroquine	Electrolyte and acid-base balance correction, steroids, CYC	Electrolyte and acid-base balance correction, steroids, CYC
Short-term neurological outcome	Response seen within 2 weeks: by 4 weeks the muscle weakness had resolved	Neurology improved within 1 month	Within a week, motor power recovered	–	Within 2/52 recovery to walking	Complicated course post-treatment – neutropenic sepsis, PE; Able to ambulate independently at 1 month	Good neurological recovery	Over several days: LOC and cranial muscle strength improved; within 2 weeks, neurology significantly improved	Improved neurology within days
Long-term neurological outcome	Occasional dizziness, 6^th^ nerve palsies persistent	Complete neurological recovery	Asymptomatic at 6 months	–	Residual right lateral rectus palsy	Recurrent relapsing disease	Persistent diplopia	Mild residual truncal ataxia	Complete neurological recovery

CPM = central pontine myelinolysis; Anti-SSA = anti-Sjogren’s syndrome-related antigen A autoantibodies; Anti SSB = anti-Sjogren’s syndrome-related antigen B autoantibodies; CYC = cyclophosphamide; IVIG = intravenous immunoglobulin; PE = plasma exchange; LOC = level of consciousness.

**Table 2. Table2:** Learning points.

Learning points in primary Sjögren’s syndrome (pSS)
pSS is a complex, multisystem disease.
Presence of systemic disease in the setting of pSS is an important prognostic determinant.
Kidney involvement accounts for ~ 5% of pSS patients.
The most common renal manifestation is tubulointerstitial nephritis, which can manifest as RTA.
In the setting of unexplained dRTA, consider pSS.
CPM is a rare neurological manifestation of pSS.
CPM is potentially fatal but can have favorable outcome if managed timeously.
Treatment with aggressive intravenous sodium bicarbonate requires careful monitoring due to the risk of CPM with rapid serum sodium changes.

pSS = primary Sjögren’s syndrome; RTA = renal tubular acidosis; dRTA = distal renal tubular acidosis; CPM = central pontine myelinolysis.
